# Genomics of CpG Methylation in Developing and Developed Zebrafish

**DOI:** 10.1534/g3.113.009514

**Published:** 2014-03-21

**Authors:** David M. McGaughey, Hatice Ozel Abaan, Ryan M. Miller, Peter A. Kropp, Lawrence C. Brody

**Affiliations:** Molecular Pathogenesis Section, Genome Technology Branch, National Human Genome Research Institute, National Institutes of Health, Bethesda, Maryland

**Keywords:** methylation, epigenetics, zebrafish, development, one-carbon metabolism

## Abstract

DNA methylation is a dynamic process through which specific chromatin modifications can be stably transmitted from parent to daughter cells. A large body of work has suggested that DNA methylation influences gene expression by silencing gene promoters. However, these conclusions were drawn from data focused mostly on promoter regions. Regarding the entire genome, it is unclear how methylation and gene transcription patterns are related during vertebrate development. To identify the genome-wide distribution of CpG methylation, we created series of high-resolution methylome maps of *Danio rerio* embryos during development and in mature, differentiated tissues. We found that embryonic and terminal tissues have unique methylation signatures in CpG islands and repetitive sequences. Fully differentiated tissues have increased CpG and LTR methylation and decreased SINE methylation relative to embryonic tissues. Unsupervised clustering analyses reveal that the embryonic and terminal tissues can be classified solely by their methylation patterning. Novel analyses also identify a previously undescribed genome-wide exon methylation signature. We also compared whole genome methylation with genome-wide mRNA expression levels using publicly available RNA-seq datasets. These comparisons revealed previously unrecognized relationships between gene expression, alternative splicing, and exon methylation. Surprisingly, we found that exonic methylation is a better predictor of mRNA expression level than promoter methylation. We also found that transcriptionally skipped exons have significantly less methylation than retained exons. Our integrative analyses reveal highly complex interplay between gene expression, alternative splicing, development, and methylation patterning in zebrafish.

The recognition of cytosine methylation in DNA predates the discovery of the structure of DNA by Watson and Crick (Wyatt 1951). By the 1980s it was recognized that although DNA sequence is largely fixed across the cells of an organism, DNA methylation varies across the genome and between cell types. More specifically, CpG methylation is tissue-specific and species-specific and correlates in some cases with transcriptional activity (Cooper 1983). By the 1990s the importance of CpG methylation in imprinted genes, which are expressed differentially on maternally and paternally derived chromosomes depending on methylation marks (Li *et al.* 1993), was clear. The recognition of this heritable non-DNA sequence–based mark has spurred the development of the study of epigenetics.

Classically, CpG methylation analysis was performed using methylation-sensitive enzymatic reactions or with bisulfite conversion coupled to Sanger sequencing (Dahl and Guldberg 2003). These methods allowed methylation states to be determined with high resolution, albeit at a severely restricted number of loci. Two newer methods can be used to score methylation at the whole genome level. The highest resolution of these is whole genome sequencing of bisulfite converted DNA. This technique allows for genome-wide coverage of methylation at base pair (bp) resolution (Frommer *et al.* 1992; Meissner *et al.* 2008). While robust, this approach is costly to apply when a study requires sequencing multiple samples. A modification of the previous approach, called reduced representation bisulfite sequencing, sequences a subset of regions adjacent to CCGG sequences (Meissner *et al.* 2008). This latter approach covers only regions of high CpG density.

We used an alternative to the techniques mentioned above, methyl-CpG binding domain protein-enriched genome sequencing (MBD-seq). MBD-seq uses methyl-CpG binding proteins or anti-methylated CpGs antibodies to purify methylated DNA from the genome. This isolated DNA is then assayed using high-throughput sequencing (Bock *et al.* 2010).

Zebrafish (*Danio rerio*) are a popular organism for the study of vertebrate development and gene function. The zebrafish genome contains approximately 26,000 genes, most of which contain several exons with large intronic space (Howe *et al.* 2013). Despite the large evolutionary distance between human and zebrafish, 70% of human genes have a clear zebrafish ortholog (Howe *et al.* 2013). The rapid early development of nearly transparent and externally fertilized embryos of zebrafish allows for relatively easy collection and drug treatment of early embryonic stages (Dooley and Zon 2000; Lawson and Wolfe 2011).

The methyl groups used in CpG methylation come from the universal methyl donor S-adenosylmethionine (SAM). SAM is a product of the one-carbon metabolic pathways that obtains its substrates from dietary, folate, and choline (Chiang *et al.* 1996; Niculescu and Zeisel 2002). The methyl groups are placed or maintained on cytosines, usually in the context of CpG in vertebrates, by DNA methyl transferase 1 (DNMT1), DNMT3a, and DNMT3b (Li *et al.* 1992; Okano *et al.* 1999). Zebrafish have orthologs to the mammalian DNMTs and deleting these genes in fish produces phenotypes that are comparable with mammals in which these genes have been disrupted (Rai *et al.* 2006, 2010). To better understand the changes that occur in the context of development, we undertook an analysis of genome-wide CpG methylation in animals at four developmental stages [sperm, one-cell, mid-blastula transition (mbt), ∼3.5 hr post-fertilization, and 3 d post-fertilization (3dpf)] and four fully differentiated (eye, brain, heart, and liver) tissues. We also disrupted one-carbon metabolism via drug treatment to see if changing the availability of one-carbon units changed the methylome. To examine the relationship between DNA methylation and gene expression, we merged our data with RNA-seq datasets derived from the same developmental stages or tissues.

## Materials and Methods

### Zebrafish stocks and sample collection

Tübingen/AB fish were used for all methylome analyses and cared for using standard practices (Westerfield 2000). Genomic DNA was collected from the sperm of 10 tricaine-killed males, more than 20,000 one-cell stage embryos, more than 10,000 mbt (∼3.5 hr post-fertilization) embryos, and 100 3dpf embryos. Embryos were staged by visual examination of anatomical markers (Kimmel *et al.* 1995). Chorions were removed with pronase for one-cell, mbt, and 3dpf. Ten adult females (∼11 months old) were tricaine-killed and dissected to collect eyes, brains, hearts, and livers. All tissues were flash-frozen with either liquid nitrogen or dry ice/ethanol, and the genomic DNA was extracted with the PureGene reagents (Gentra). Bisulfite conversion for specific loci analysis was performed using the EZ DNA Methylation Kit (Zymo) per the manufacturer’s protocol.

### Methotrexate treatment

We applied 400 µM methotrexate (MTX; NDC 63323-123-10 102310; APP Pharmaceuticals) to one-cell zebrafish in E3 embryo medium. One hundred of these MTX-treated zebrafish were raised to 3dpf. Genomic DNA was extracted from these embryos as reported above.

### Methylation enrichment

Genomic DNA was sonicated to 300-bp fragments with a Covaris S2 with the following settings: 10% duty cycle; 4 intensity; and 200 cycles/burst for 90 sec in 130 µl Tris-EDTA. No more than 10 µg gDNA was used per sonication reaction. DNA was concentrated in a Speed Vac SC110 A. Methylation enrichment was performed using EpiMark DNA Enrichment kit (New England Biolabs). Both the captured (methylated) DNA and flow-through (unmethylated) DNA were retained for downstream analysis. The degree of enrichment was assessed with SYBR quantitative PCR on the Applied BioSystems 7900HT with both the DNA Methylation control package (EF-100-0040; Diagenode) and custom primers designed to amplify the zebrafish *tert* and *sox2* loci, previously reported to be methylation-rich and methylation-poor, respectively (Lindeman *et al.* 2010). The methylation enrichment was calculated by comparing the Ct value of the methylated loci to the unmethylated loci in the captured DNA and the flow-through DNA (Supporting Information, Table S1).

### Sequencing

Multiplex libraries were created using ChIP-Seq DNA Sample Prep Kit (Illumina) and sequenced with 101-bp paired-end reads with an Illumina HiSeq 2000.

### Bioinformatics analysis

Illumina Fastq files were aligned to the zebrafish Zv9 build using BWA (Li and Durbin 2009). See Table S2 and Figure 2 for read information. Only properly paired reads with mapQ >5 were used. Peaks were called using MACS2 2.0.10.20120703 (Zhang *et al.* 2008). Through the use of technical replicates, we found that variation in peak width was driven more from experimental factors than biological variation. In contrast, peak location was highly reproducible. As such, we used the centers of the peaks and expanded them by 100 bp in each direction for our analyses. Raw genome-wide histone localization data were obtained from Ulitsky *et al.* (2011) and the reads were aligned with BWA and peaks were called with MACS2. RNA-seq datasets for one-cell and mbt are under accession number SRX025029. The 3dpf RNA-seq datasets used combined data from accession numbers SRR065196 and ERX008921. Brain RNA-seq datasets used combined data from accession numbers ERX009448 and ERX013540. Heart RNA-seq datasets used combined data from accession numbers ERR023145 and ERR023145. Liver RNA-seq datasets used combined data from accession numbers SRR392106 and SRR392106.

Illumina-based sequencing reads were aligned with STAR 2.1.2 (Dobin *et al.* 2013) against the Zv9 zebrafish build. ABI-based sequencing reads were aligned with LifeScope 2.5.1. mRNA transcription scores were calculated, making counts with HTSeq and calculating RPKM with cqn correction in R (Anders 2010; Hansen *et al.* 2012). Running analyses without cqn correction yields similar results.

The meta-gene was created using custom Unix, Python, and R scripts as well as BEDTools and ggplot2 (Quinlan and Hall 2010; *ggplot2 - Elegant Graphics for Data Analysis*). Briefly, ensembl68 zebrafish gene annotations were used to identify exons, introns, and UTRs. In cases where there were multiple overlapping features, *i.e.*, overlapping exons with slightly different stop and start positions, the feature was expanded to encompass all annotations. Promoters were defined as 2000 bp 5′ of the transcriptional start site. Any promoters that overlapped another annotated gene were removed. The upstream and downstream regions were defined as 10,000 bp upstream and downstream of the promoter and 3′ UTR, respectively. Any upstream or downstream region overlapping an annotated gene was removed. Exons and introns were split into quarters and thirds by position in the gene, respectively. Methylation ratio scores were calculated in 20 windows per element (see below). The features with their subdivided windows were then averaged across the entire genome and plotted.

Methylation enrichment is calculated by comparing the peak/feature intersection numbers to peak/feature intersections where the peak positions are randomized by BEDTools shuffle 1000 times. This provides the enrichment of methylation compared with a situation in which the peaks are randomly distributed. The 1000-fold bootstrapping procedure allows us to calculate a standard deviation for these values.

The methylation ratio score is similar to an RPKM score and is calculated with the following equation: (number of methylation-enriched sequencing reads in window/total methylation-enriched sequencing reads) / (number of methylation-unenriched reads in window/total methylation-unenriched reads). The windows are 5% of the length (in bp) of the feature. We found that correcting for CpG content did not improve the concordance of methylation scoring between biological or technical replicates.

The aligned reads were used by ChromHMM to determine two states: methylated or unmethylated for 200-bp windows across the genome (Ernst and Kellis 2012). The ChromHMM makesegmentation script was used to output the posterior probabilities for methylation at each 200-bp window for the different tissues. The posterior probabilities were used by kmeans clustering in R to create a heatmap of methylation relationships between the tissue types. The clustering was performed more than 12 times, demonstrating that 14 clusters were found to provide the most reproducible and consistent organization of clusters across the different tissue types.

Spliced-out exons were identified by using splice junctions detected by STAR and intersecting them with Ensembl gene models. More specifically, custom Unix and Python scripts were used with BEDTools to identify cases where the 5′ and 3′ ends of the splice junctions are in different exons. If there is a single annotated exon between the aligned exons, then this is declared a skipped exon. Peaks were intersected against the identified splicing exon/intron classes. Enrichment was calculated by taking the results of bootstrapping 1000 fold with position-randomized peaks and comparing against the original peaks. The SD was calculated from the distribution of the 1000 fold bootstrapping.

### Data

Peak files and raw sequencing files are available under GEO accession GSE52110.

## Results

### Genome-wide methylation patterning of zebrafish is similar to humans

To study the methylomes of developing and developed zebrafish, we purified DNA from cells and tissues by first independently pooling the sperm of 10 males, 20,000 one-cell embryos, more than 10,000 mbt embryos, and 100 3dpf embryos, and the eyes, brain, hearts, and livers of 10 females. Methylation-rich DNA regions were selected and the methylation-enriched fraction (with no NaCl extraction) and the methylation-poor flow through were sequenced with an Illumina HiSeq 2000. More than 10 million unique properly paired reads were generated for each sample (Table S1).

To independently assess the MBD-seq technique, we compared our data with those obtained from whole-genome bisulfite sequencing. We downloaded whole-genome bisulfite sequencing data derived from mbt stage embryos (GSM1133397) (Jiang *et al.* 2013). Read coverage and methylation ratio scores (see *Materials and Methods* for methylation ratio calculation) from these data were compared with our MBD-seq data from the same developmental stage. We found a substantial concordance between the two techniques. At least one read from our methylation-enriched regions covered 70% of called methylated CpGs and the methylated CpGs called by Jiang *et al.* (2013) had a significantly higher MBD-seq methylation ratio score than their unmethylated CpGs (Figure S1).

**Figure 1:**
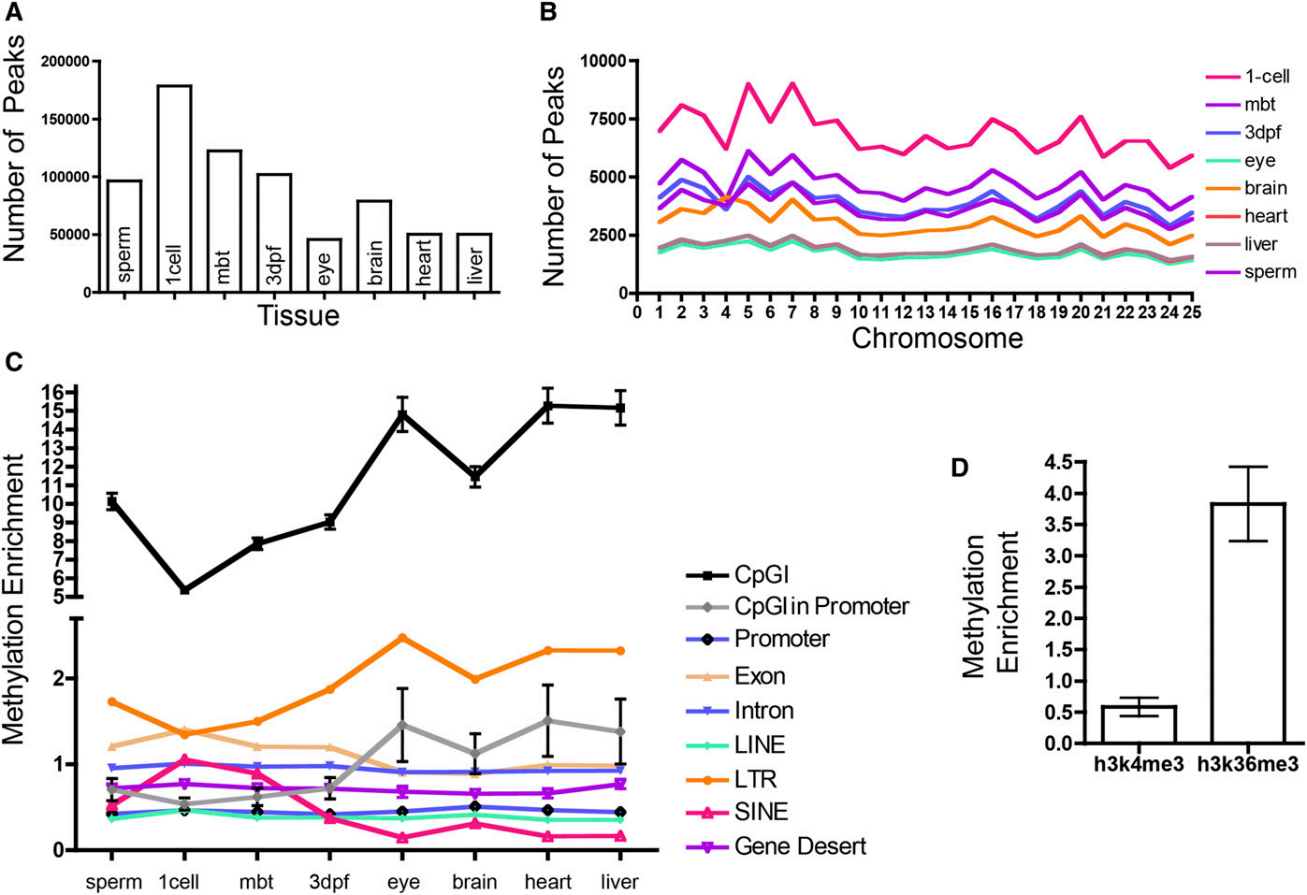
Distribution of highly methylated regions (peaks) across the genome among different zebrafish tissues. (A) The number of MACS2 called peaks in the eight different tissues assayed. (B) The number of peaks in the eight tissues in each of the 25 different zebrafish chromosomes. (C**)** The enrichment of methylation peaks relative to randomly distributed peaks across common genomic elements. All values have *P* < 0.001, except for 3dpf Intron (*P* = 0.15). Error bars show SD. (D) Enrichment of methylation peaks in 3dpf zebrafish at h3k4me3 (*P* = 0.013) and h3k36me3 (*P* < 0.001) histone marks. Error bars show SD (not shown where error bars are smaller than symbol).

The MACS2 peak caller was used to identify highly methylated regions (Zhang *et al.* 2008). The sample with the fewest peaks (45,785) was the eye, and one-cell embryos had the most peaks (178,587) (Figure 1A). These peaks are evenly spread across the zebrafish chromosomes (Figure 1B). The peak caller also provides magnitude scores for each peak. To confirm enrichment for methylation and to see whether the score correlated with methylation, we examined numerous bisulfite converted regions in 3dpf zebrafish embryos using Sanger sequencing (Figure S2). This confirmed that peak regions are highly methylated, relative to non-peak regions. However, we noted that there was no relationship between the peak “score” generated by MACS2 and the amount of CpG methylation.

The distribution of peaks across the zebrafish was overlaid on genomic features taken from UCSC Zv9 build and Ensembl-annotated genes. Enrichment was calculated by using a bootstrapping approach (Figure 1C) (see *Materials and Methods*). In all tissues, peaks are highly enriched across CpG islands, in contrast to the findings of Lister *et al.* (2009) in human samples. These peaks cluster more tightly across the CpG islands of the terminal tissues. It has been suggested that CpG island prediction algorithms are inaccurate in non-mammalian vertebrates and provide an experimentally derived non-methylated island (NMI) set as a substitute for CpG islands for the zebrafish (Long *et al.* 2013). Consistent with the data from Long *et al.* (2013), we also found significant under-enrichment of our peaks in NMIs (approximately half as many peaks as expected by chance; data not shown). This result may explain our disparate CpG island methylation findings in zebrafish as compared to the findings in humans by Lister *et al.* (2009).

There are more than 12,000 CpG islands in the Zv9 zebrafish genome, as defined by UCSC; 1411 of these islands overlap promoters. We found that, as expected, CpG islands in promoters are slightly methylation-poor in the embryonic tissues. Terminal tissues differ slightly in their CpG methylation patterning. In the terminal tissues, CpG islands in promoters, although far less methylated than CpG islands overall, are still slightly methylation-rich.

In zebrafish, promoter regions, defined as 2000 bp upstream of annotated genes, are methylation-poor, similar to humans and other species (Feng *et al.* 2010). Ensembl-annotated exons and introns show little enrichment of methylation. We note that gene deserts, regions more than 250,000 bp from annotated genes, are slightly methylation-poor in all tissues, suggesting that methylation is being targeted toward gene-rich areas. Because variation in local GC or CpG content could drive these differences, we calculated GC and CpG content of gene deserts and exons. We found that the density of these dinucleotides is approximately the same in gene deserts and exonic regions (data not shown).

To see whether histone modifications that are important in transcription correlate with methylation patterning, we examined the relationship between H3K4me3 and H3K36me3 histone marks using 3dpf zebrafish ChIP-seq data from Ulitsky *et al.* (2011). H3k4me3 is associated with promoters and active enhancers, whereas h3k36me3 is associated with transcriptionally active gene-bodies (Kouzarides 2007; Wang *et al.* 2008). We found weak methylation signal in the former and strong methylation signal in the latter. These results match previously published results in human cells (Hawkins *et al.* 2010).

### LINEs and SINEs have different methylation patterns in embryonic and terminal tissues

Because methylation of repetitive elements is known to be dynamic during development, we examined the distribution of peaks in three major classes of repetitive elements: LINEs, SINEs, and LTRs (Smith *et al.* 2012). We found that these three classes have distinct patterns across the different tissues, with LTRs and SINEs having differential enrichment in embryos compared to terminal tissues. LTRs are methylation-rich in all tissues, with the terminal tissues having stronger enrichment, whereas SINEs have the opposite pattern with low methylation signal overall and even lower signal in the terminal tissues. LINEs are methylation-poor in all tissues and do not show the embryonic/terminal differential methylation signal.

### Unsupervised clustering reveals that genome-wide methylation signals can reliably distinguish tissues

**Figure 2:**
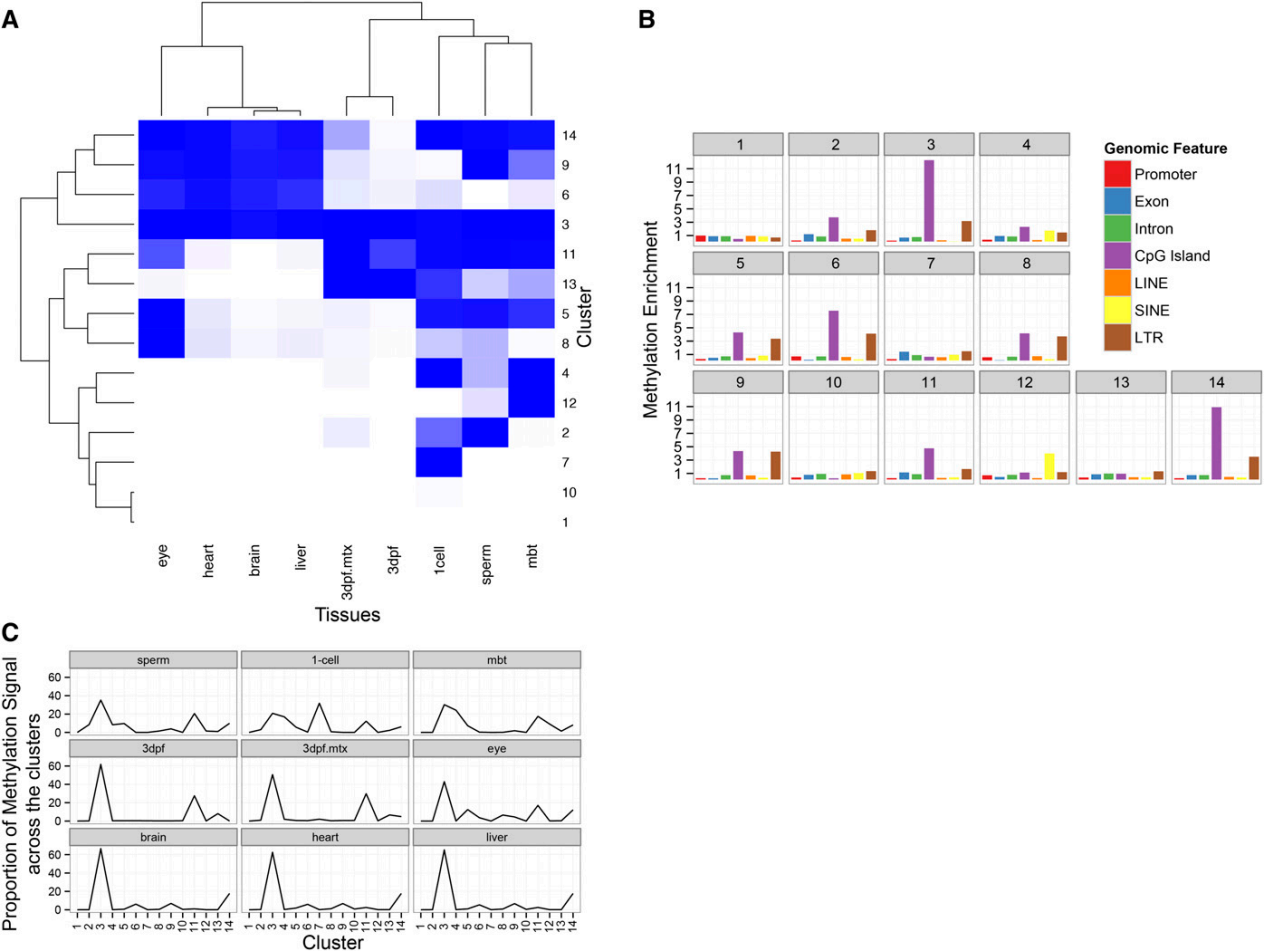
Unsupervised clustering of methylation signal reveals relationships shared and unique methylation patterning between embryonic and terminally differentiated tissues. (A) K-means clustering of the genomic locations with ChromHMM calculated probability of methylation status. Each cluster contains genomic coordinates. Darker blue indicates higher methylation levels. The length of the branches in the dendograms illustrates the relatedness of the clusters and tissues. (B) Each cluster was assayed to determine which genomic features are over-represented in each cluster. Enrichment is calculated as described previously by comparing the features overlapping cluster genomic coordinates relative to randomly distributed coordinates. (C) Proportion of methylation signal in each cluster, calculated by adding the number of methylated regions assigned to each cluster.

To analyze genome-wide methylation patterning in a more agnostic manner, we used ChromHMM on the filtered sequencing reads to identify methylation-positive and methylation-negative states (Ernst and Kellis 2012). Those states were clustered with an unsupervised k-means algorithm (see *Materials and Methods*). We found 14 clusters to create the most stable divisions of the data (Figure 2A). Of note, these clustering algorithms produce clusters that segregate embryonic and terminal tissues. The 3dpf tissue type is a partial exception to this rule. Approximately half of the time the k-means clustering placed this stage with the terminal tissues (data not shown).

To better understand the relationships between the different tissues, the cluster data were further subdivided to resolve genomic features overlapping the coordinates of each cluster (Figure 2B). Clusters 1 and 10, which have no or little methylation signal, have enrichment of no particular genomic feature. In contrast, cluster 3, which has the genomic coordinates where all tissues are methylated, is enriched for CpG islands and LTR. Cluster 14, which is similar to cluster 3 except that the 3dpf tissues are not highly methylated, has a similar pattern.

### Most segments of methylated DNA are in similar positions across the zebrafish tissues

The clustering also allows us to see how methylation patterns are shared across the tissues. Most prominent is that the majority of the methylation signal is at the same positions (Figure 2C, cluster 3) in the genome across the different tissues. The terminal tissues, brain, heart, and liver, have more than 60% of their methylation signal in cluster 3, whereas the eye has more than 40%. With the exception of the one-cell stage, the majority of methylation in embryonic tissues is associated with cluster 3. The embryonic tissues share more than 15% of their methylation signal in cluster 11, which is enriched for CpG islands. The one-cell stage has a unique set of signals in cluster 7. However, this cluster does not have any unique enrichment in genomic features or GO terms (data not shown).

### Compressing genome-wide methylation data into the meta-gene reveals asymmetric methylation across genes

**Figure 3:**
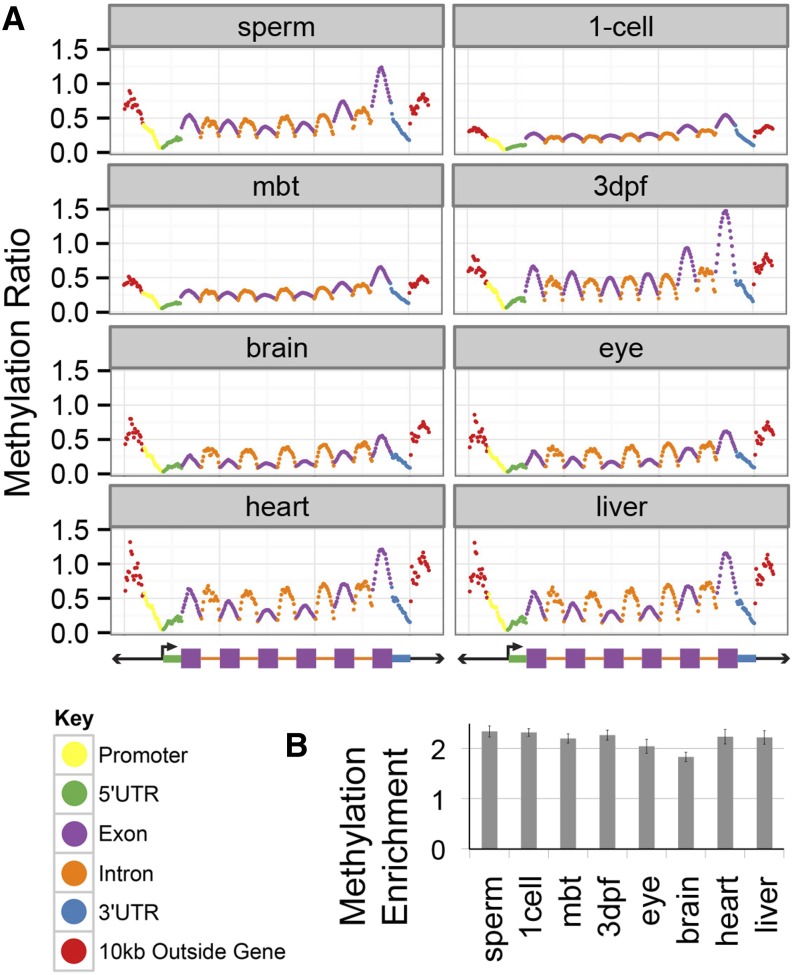
The meta-gene reveals unique methylation patterning across gene elements in the zebrafish genome. (A) The relative methylation of the gene elements was calculated in 20 bins per feature and then averaged for all gene elements in the genome. Exons and introns were further subdivided by first, last, and quartiles by their relative position in a given gene. (B) Methylation enrichment was calculated by a different method: relative enrichment of peaks in the last exons over chance. This demonstrates the strong enrichment of methylation in the last exons of genes. All enrichments differ from randomly distributed peaks (empirical *P* < 0.001). Error bars show SD.

To assess whether methylation was differentially positioned in the “average” gene, we overlaid our data onto a representative “meta-gene” (Figure 3A). The meta-gene was created by first parsing each Ensembl gene into several elements: 10,000 bp upstream and downstream of the genes; promoters; and 5′ and 3′ UTR. Because the average zebrafish gene has approximately four exons, we distributed exons into quarters by position across the gene flanked by the first and last exons and calculated methylation ratio in windows (see *Materials and Methods*).

This visualization of methylation reveals patterns shared across all of the tissues (Figure 3A). First, as has been previously reported, methylation decreases across the 5′ UTR and increases after the transcriptional start site (Feng *et al.* 2010). Second, the last exons tend be more methylated than interior exons. Overall, the last exons are approximately two-fold enriched for methylation (Figure 3B). This pattern was consistent in all embryonic stages and terminal tissues. Exons and introns show a characteristic ∩ shape, which is reminiscent of methylation patterning in humans and invertebrates (Lister *et al.* 2009; Nanty *et al.* 2011; Gelfman *et al.* 2013). The relative levels of methylation between introns and exons are approximately the same in the embryonic tissues while the introns tend to be more methylated than the exons in the terminal tissues. As has been previously reported, methylation gradually increases across the gene 5′ to 3′ (Feng *et al.* 2010).

### Comparisons of methylation and transcriptional data sets demonstrate that promoter methylation is not the strongest determinate of gene expression

**Figure 4:**
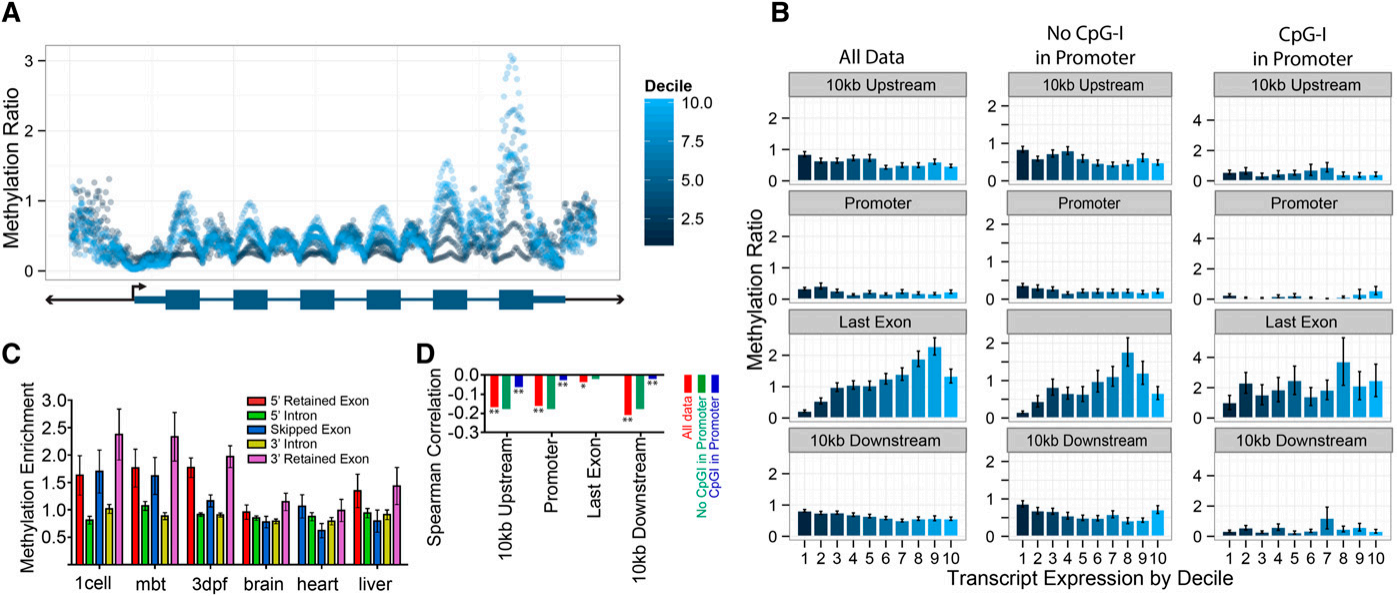
Gene-body methylation is correlated with transcriptional levels and alternative splicing. (A) The metagene for 3dpf zebrafish is split into deciles, by transcription, measured by RPKM (with 10 being the highest expressing decile). (B) The 3dpf zebrafish methylation ratio for the entire element is split by transcription expression deciles (with 10 being the highest expression decile). The data are further split into two groups: one showing the effects of only considering genes where there is a CpG island in the promoter and a second set of genes with promoters lacking CpG islands. (C) Skipped exons were identified and the methylation enrichment was plotted for the skipped exon and the surrounding introns and retained exons. Error bars show SE. (D) Spearman correlations for 10 kb upstream and downstream, promoter and last exon, further split by presence or absence of a CpG island in the promoter (**P < 10^−25^; *P < 10^−7^). Error bars show SE.

To study the relationship between gene transcription and methylation, we used publicly available RNA-seq datasets for wild-type zebrafish one-cell, mbt, and 3dpf embryos and for brain, heart, and liver (Rösel *et al.* 2011; Yang *et al.* 2012; Aanes *et al.* 2011) (see *Materials and Methods*). We reprocessed the raw RNA-seq data to quantify gene transcription and to identify splicing events (see *Materials and Methods*). We divided the meta-gene into deciles by gene transcription levels to determine if differentially expressed genes had unique methylation patterns (Figure 4A). Because only genes with associated promoters were used, approximately 5000 out of the approximately 17,000 genes were not used in these analyses. We found that the methylation and expression were most strongly correlated not at the promoter, as has been widely reported, but with regions 10,000 bp upstream and downstream from the gene. We also observed that while the last exons show a dramatic positive trend between increasing methylation and increasing transcription, the magnitude of the spearman correlation when assessing all genes is not very strong (Figure 4D).

Work in other organisms has demonstrated that the presence of CpG islands in the promoter disrupts the relationship between expression and promoter methylation (Varley *et al.* 2013). We found a similar result, with the correlation between promoter and methylation and transcription weakening if there is a CpG island in the promoter. Interestingly, we also found that the negative correlation between methylation and transcription in the upstream/downstream regions is also diminished when a CpG island is present in the promoter (Figure 4B).

### Skipped, alternatively spliced exons have differential methylation levels

Because work by Shukla *et al.* (2011) mechanistically links DNA methylation to exon splicing, we asked if zebrafish genome methylation has a relationship to splicing. We first identified skipped exons in the six RNA-seq datasets and compared methylation enrichment at the skipped exon with the surrounding retained exon and intervening introns (Figure 4C). We found that embryonic and terminal tissues have different methylation patterns around spliced out exons, with the embryonic tissues having stronger methylation enrichment in the exons relative to the introns and the terminal tissues having similar methylation between the introns and exons. Between the two types of exons (skipped and retained), we note that in the embryonic tissues there is not a large difference in methylation between the two types. In contrast, the terminal tissues have a more distinct pattern, with the skipped exons having lower methylation levels than the retained exons.

### Methotrexate treatment reduces overall methylation levels and subtly influences gene-specific methylation patterns

MTX is a potent inhibitor of the dihydrofolate-reductase enzymatic conversion of dihydrofolate to tetrahydrofolate. The block of this reaction prevents the conversion of homocysteine to methionine, a necessary molecule for the creation of the universal methyl donor s-adenosyl methionine. Application of MTX to early zebrafish embryos results in shortened anterior–posterior axis, cardiac defects, perturbed cell cycles, and highly increased mortality (Lee *et al.* 2012). We treated one-cell embryos with MTX and collected DNA at 3dpf. Methylation was analyzed in the same manner as before (see *Materials and Methods*).

**Figure 5:**
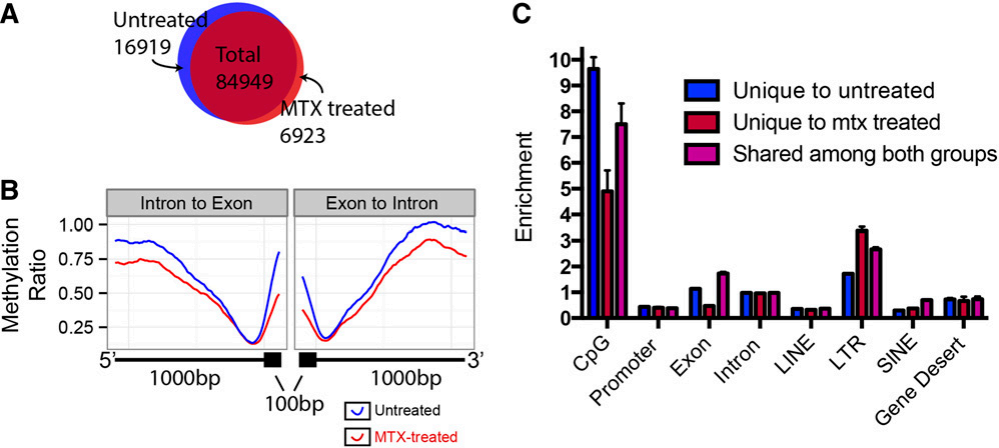
Methotrexate (MTX) treatment of 3dpf zebrafish reduces genome-wide methylation in specific regions. (A) Methylation peak locations were compared between the untreated and MTX-treated 3dpf zebrafish samples. (B) Methylation ratio of 1000 bp of an intron and 100 bp of the adjacent exon, averaged for the entire genome. Untreated 3dpf in blue, MTX-treated 3dpf in red. (C) Enrichment of methylation peaks for peaks unique to the untreated 3dpf (blue), unique to MTX-treated 3dpf (red), and shared (purple) among common genomic elements. CpG refers to CpG islands. Error bars show SD.

There are three probable outcomes from the MTX treatment in genome-wide methylation patterning. First, there may be no significant difference in methylation. Second, methylation could be reduced globally. Third, methylation intensity could be different at specific genomic positions. We found that genome-wide methylation patterning is largely identical between MTX-treated 3dpf and untreated 3dpf zebrafish (Figure 2A, Figure 5A). There are some subtle differences. The MTX-3dpf zebrafish have several thousand fewer detected methylation peaks (Figure 5A) consistent with reduced availability of methyl donors. The MTX-3dpf zebrafish have lower methylation in introns and exons (Figure 5B). We identified peaks that, among all 3dpf peaks, were unique to MTX treatment and unique to the no-treatment set. The MTX treatment–unique peaks, compared with the peaks unique to no-treatment, are much less likely to be in CpG islands and exons and more likely to be in LTRs (Figure 5C). GO term analysis of the genes with unique peaks in exons of the MTX-treated set with WebGestalt reveal that there is a weak enrichment of the molecular functional category methyltranferase activity, consistent with the role of MTX in the creation of SAM (Figure S3) (Zhang *et al.* 2005).

## Discussion

### Methylation across the zebrafish genome is unevenly distributed

Despite a long history of studying DNA methylation, only recently has it become possible to analyze, in high-resolution, DNA methylation across the genome. Here, we successfully performed MBD-seq covering the entire zebrafish genome in eight different embryonic tissues and stages and examined the influence of a drug that interferes with the methyl donor producing pathway. At the macro level, methylation is evenly distributed across the 25 chromosomes of the zebrafish. While appearing to be uniform when averaged over long distances, methylated CpGs are not randomly spread across the genome. As expected, methylation density is concentrated in CpG islands and moderately enriched in LTRs. In contrast, LINEs and promoters have lower methylation signals. We also note that terminal tissues and embryonic tissues have different proportions of methylation across the genomic elements, which can be seen most clearly in LTRs, SINEs, and CpG islands.

### Clustering reveals unique methylation signatures for each tissue and corroborates whole-genome bisulfite sequencing analyses

Our unsupervised clustering analysis allows us to distinguish between embryonic and terminal tissues using global methylation patterns alone, which demonstrate that each tissue or time point carries a unique methylation signature. In addition, the distribution of methylation across the clusters produced highlight two trends. First, the majority of the genome-wide methylation signal assayed is shared in a common set of genomic coordinates in the embryonic tissues and a majority in the terminal tissues. Second, the embryonic tissues have more diverse and widespread methylation signal, suggesting that as tissues differentiate CpG methylation is selectively removed or lost.

Publications by Jiang *et al.* (2013) and Potok *et al.* (2013) have recently assessed genome-wide methylation patterning with whole-genome bisulfite sequencing. These authors found that the maternal methylation state predominates after fertilization until approximately the mbt stage, when the embryo more closely resembles the sperm methylome. While we did not evaluate the oocyte methylome, our clustering places the sperm methylome most closely with the mid-blastula stage, in agreement with the results of Jiang *et al.* (2013) and Potok *et al.* (2013).

### Meta-gene analysis demonstrates that gene-body methylation is stable across time, but unique among different elements of the gene

We created the meta-gene to represent the methylation anatomy of the “average” gene. These analyses reveal a previously undescribed pattern of methylation signals across the average gene. Specifically, last exons appear to contain a disproportionate amount of methylation. The meta-gene also demonstrates that across the tissues and time points assayed, that gene-body element methylation patterning is largely stable. The only notable difference between embryonic and terminal tissues is that exon methylation is a bit lower compared to intron methylation as the tissues develop.

### The relationship of CpG methylation with transcription is not limited to promoter methylation

Because CpG methylation is classically reported as influencing gene transcription, we merged published RNA-seq datasets with our genome-wide methylation data. This allowed us to probe the relationship between gene-body methylation and transcriptional levels. In contrast to expectation, we found that promoter methylation is not the strongest determinate of transcription levels. Methylation signal in the last exon of a gene is a better predictor than promoter methylation, with increasing methylation associated with higher transcript levels. We note that regions upstream and downstream of genes show stronger correlation between methylation and transcriptional levels than promoter methylation. We also examined the relationship between DNA methylation and patterns of alternative exon splicing. This revealed that methylation status of alternatively spliced exons differs from that of constitutively expressed exons. This observation held true for embryonic and terminal tissues, suggesting that different processes are used to mark spliced out exons in differentiated tissues.

### The influence of methoxtrexate on zebrafish methylation levels and patterning suggest a model for its chemotherapeutic effects

MTX is used in humans as a chemotherapeutic agent and in low doses to treat rheumatoid arthritis. It is a strong inhibitor of the DHFR, which catalyzes the conversion of DHF to THF. This has a major effect on the nucleotide pools available for DNA synthesis. This inhibition is also predicted to reduce the number of methyl donors potentially available for DNA methylation reactions. Previous research has demonstrated that this drug also disrupts zebrafish development. We found that it appears to reduce methylation across the genome, with the strongest effects happening at CpG islands and exons. This suggests that a downstream effect of this drug, besides its known role in nucleotide metabolism, may be to disrupt the methylation patterning of important genome elements and proteins, potentially disrupting transcriptional and protein activity.

Our analysis of methylation patterning in zebrafish reveals that this important model organism has similar canonical methylation patterning as other vertebrates and mammals.

## Summary

In this study, we leverage our datasets against other ChIP-seq and RNA-seq datasets to demonstrate the complexity of methylation patterning in relation to histones, alternative splicing, and transcription. Analysis of disparate genome-wide datasets allows us to make generalizations about the relationships between epigenetic modifications and gene expression, which especially demonstrates the need to re-evaluate the emphasis on promoter methylation being important in modifying gene expression. Our novel meta-gene analysis provides a useful template for analyzing the effects of methylation changes on the gene-body across the genome and highlights how methylation changes across the gene. Further research is needed to determine whether methylation is driving these processes or is a passenger mark. Our work confirms that methylation is a dynamic process. It also highlights the power of using genome-wide analyses, without which the importance of DNA methylation outside of gene promoters would remain hidden from view.

## 

## Supplementary Material

Supporting Information
