# Supplementary material for: Genomics of CpG Methylation in Developing and Developed Zebrafish
Source: G3 (Bethesda). 2014 Mar 21;4(5):861–9. doi: 10.1534/g3.113.009514 (PMC4025485; doi:10.1534/g3.113.009514)
Supplement: Supporting Information [file supp_4_5_861__index.html]

Genomics of CpG Methylation in Developing and Developed Zebrafish — Supporting Information 

# Genomics of CpG Methylation in Developing and Developed Zebrafish

## Supporting Information for McGaughey *et al.*, 2014

**Files in this Data Supplement:**

- Supporting Information - Figures S1-S4 and Tables S1-S3 (PDF, 1 MB)
- Figure S1 - Concordance of MBD-seq and Jiang *et al.* whole genome bisulfite-seq (WGBS) at the MBT time point in zebrafish. (PDF, 158 KB)
- Figure S2 - MACS2\_score versus bisulfite-converted CpG methylation (sanger sequencing). (PDF, 162 KB)
- Figure S3 - WebGestalt molecular function GO term analysis genes with unique exon peaks in 3dpf methotrexate treated zebrafish relative to control 3dpf zebrafish. (PDF, 334 KB)
- Figure S4 - Clustering of RNA-seq expression across the six cell types. (PDF, 1 MB)
- Table S1 - Enrichment performance of the NEB EpiMark 5-mC system. (PDF, 128 KB)
- Table S2 - Enrichment performance of the NEB EpiMark 5-mC system. (PDF, 128 KB)
- Table S3 - Primer sequence and coordinates for primers used in Table S2. (PDF, 127 KB)
