# Supplementary material for: Genomics of CpG Methylation in Developing and Developed Zebrafish
Source: G3 (Bethesda). 2014 Mar 21;4(5):861–9. doi: 10.1534/g3.113.009514 (PMC4025485; doi:10.1534/g3.113.009514)
Supplement: Supporting Information [file supp_g3.113.009514_009514SI.pdf]

G3·Genes | Genomes | Genetics

Genomics of CpG Methylation in Developing and Developed Zebrafish

David M. McGaughey[1], Hatice Ozel Abaan[1], Ryan M. Miller[1], Peter A. Kropp [1], Lawrence C. Brody[1]

[1]Molecular Pathogenesis Section, Genome Technology Branch, National Human Genome Research Institute, National Institutes of Health, Bethesda, MD, USA.

Corresponding Author:
Lawrence C. Brody, Ph.D.
Building 50, Room 5306
50 South Drive
Bethesda, MD 20814
301-496-7824
lbrody@mail.nih.gov

Data Deposit:
GSE52110

DOI: 10.1534/g3.113.009514

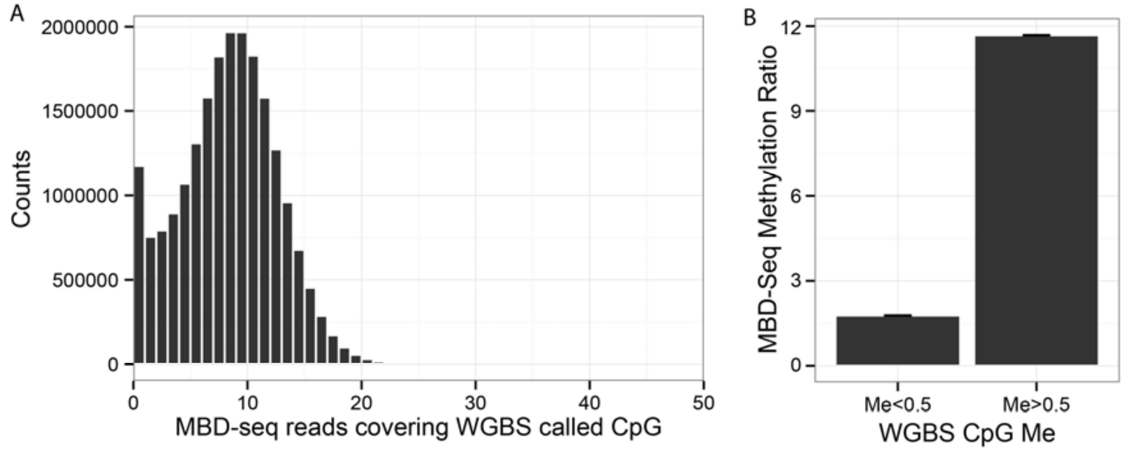

Figure S1 Concordance of MBD-seq and Jiang *et al.* whole genome bisulfite-seq (WGBS) at the MBT time point in zebrafish. (A) Histogram of methylation enriched reads overlapping Jiang et al. identified methylated CpGs. Over 70% of the $19.4x10^6$ CpGs with a methylation of greater than 0.5 (1 being fully methylated, 0 being unmethylated) have at least one overlapping MBD-seq read. (B) Average MBD-seq read ratio scores at Jiang et al. CpGs with methylation less than 0.5 and greater than 0.5. Standard error shown.

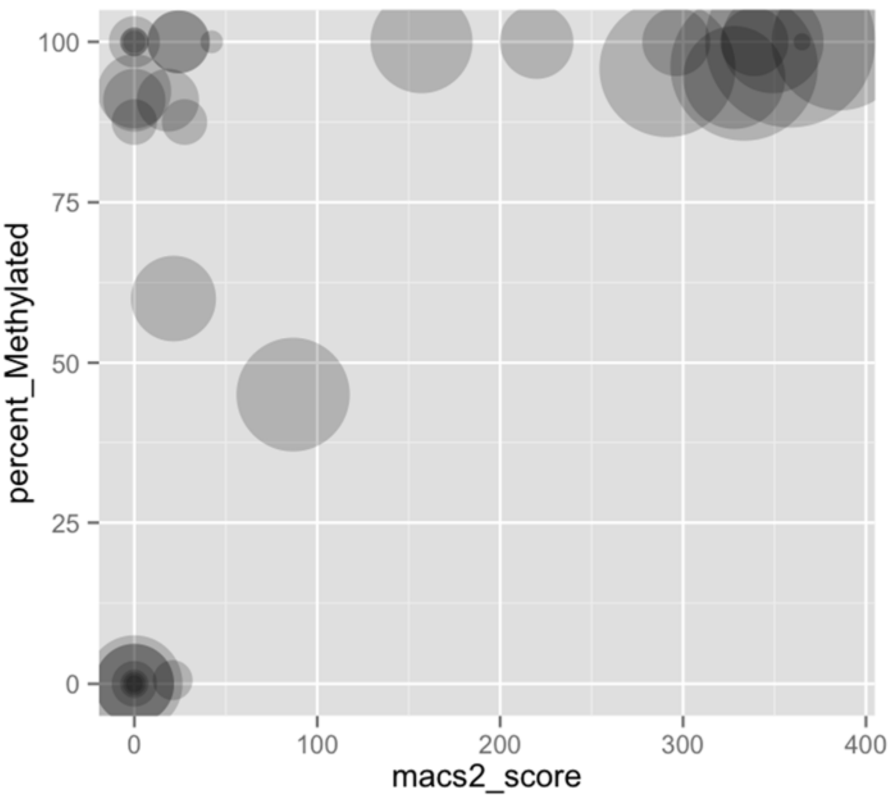

Figure S2 MACS2_score versus bisulfite-converted CpG methylation (sanger sequencing). MACS2 score is on the x-axis with percent CpG methylation on the y-axis, as determined by bisulfite conversion and sanger sequencing. The size of the dot is proportional to the number of CpGs assayed.

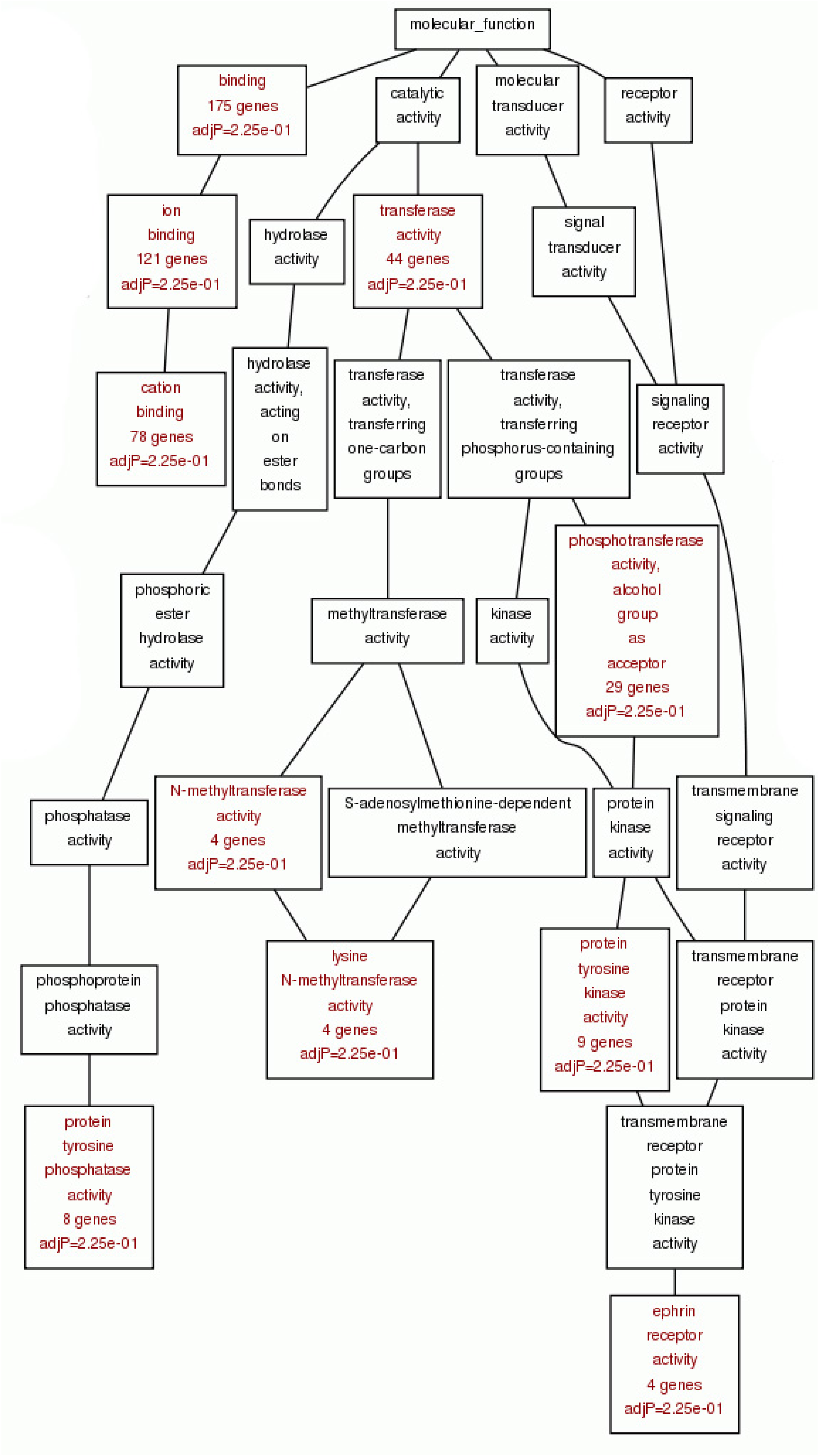

Figure S3 WebGestalt molecular function GO term analysis genes with unique exon peaks in 3dpf methotrexate treated zebrafish relative to control 3dpf zebrafish.

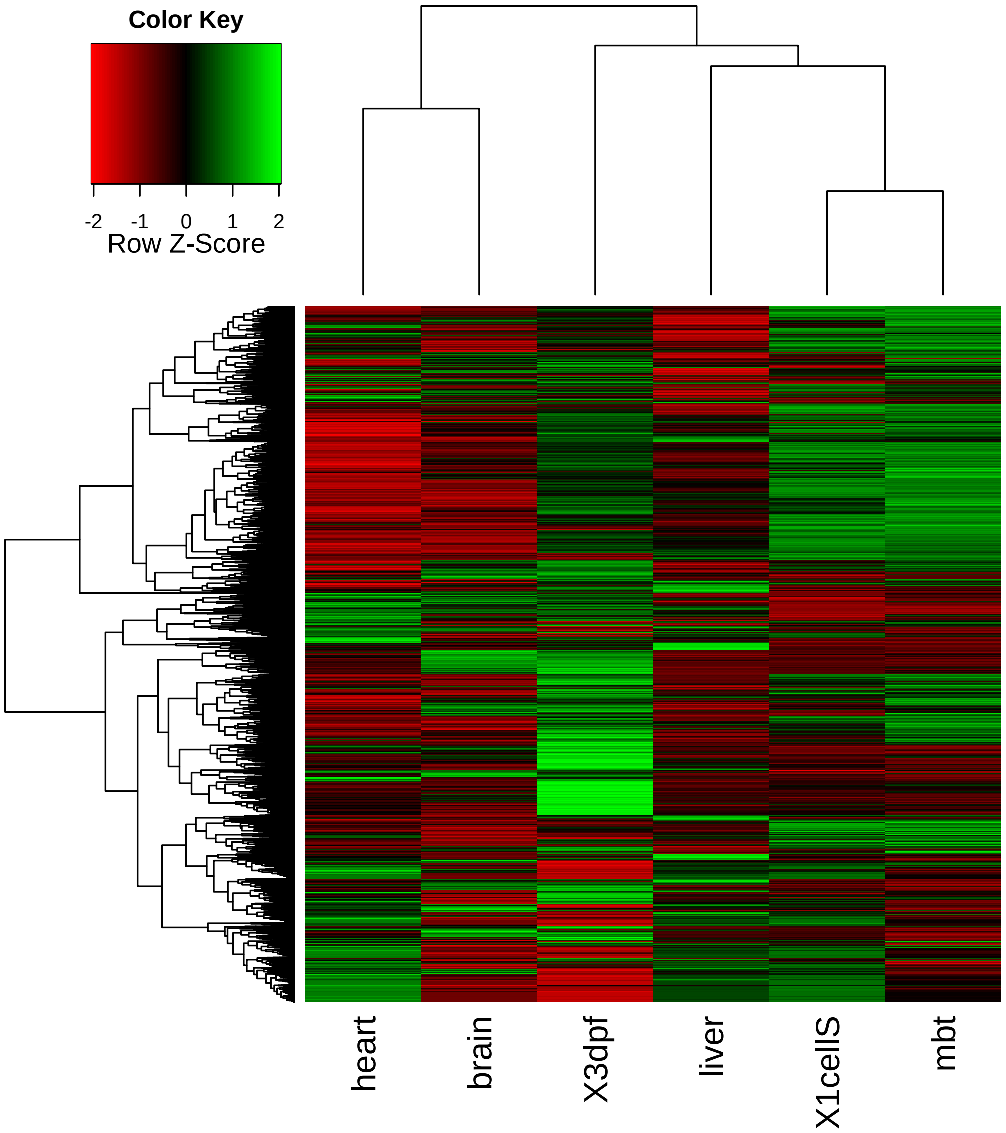

Figure S4 Clustering of RNA-seq expression across the six cell types. All zebrafish genes are clustered on the rows.

Table S1 Enrichment performance of the NEB EpiMark 5-mC system. Ct scores from qPCR are shown along with the standard deviation. Enrichment is calculated by comparing Ct values from regions known to be methylation positive (me+) to regions known to be methylation poor (me-).

Lane	Tissue	Number of Total Reads	Number of Reads mapQ > 5	Number of Properly Paired Unique Reads, mapQ > 5
4	Brain	87,411,962	40,717,029	17,146,152
4	UnMe Brain (Control)	93,316,244	74,579,958	37,140,721
4	Liver	89,493,944	43,088,933	18,221,225
4	Heart	88,729,694	42,024,288	18,593,167
4	Eye	86,522,756	39,303,576	14,639,460
5	Brain	89,582,450	41,089,919	17,344,084
5	UnMe Brain (Control)	89,959,638	71,824,799	35,933,160
5	1-cell	69,856,440	45,189,947	15,677,629
5	Sperm	75,491,368	42,197,998	19,429,347
5	MBT	63,402,436	36,063,223	17,375,340
3	3dpf.mtx	86,531,596	49,655,034	16,627,123
3	3dpf	85,494,066	45,460,989	19,458,515
3	3dpf.mtx UnMe (Control)	95,881,946	76,243,106	38,340,957
3	3dpf UnMe (Control)	95,881,946	89,661,095	45,038,786

Table S2 Enrichment performance of the NEB EpiMark 5-mC system. Ct scores from qPCR are shown along with the standard deviation. Enrichment is calculated by comparing Ct values from regions known to be methylation positive (me+) to regions known to be methylation poor (me-).

	Me_Enriched		Flow_through (me_poor)	
primers	Ct score	stdev	Ct score	stdev
tert (me+)	21.1439275	0.171582996	22.16167	0.003125412
sox2 (me-)	32.9350965	0.115988847	21.146596	0.331689649
diagenode spike me+	21.6001225	0.356402324	21.750327	0.09125213
diagenode spike me-	32.073724	0.252106195	19.2906445	0.09318041
Enrichment (in Ct)				
tert/sox2	12.806243	0.391052335		
diagenode spike set	12.933284	0.455620116		
Fold Enrichment:				
tert/sox2	5461.913175	to	9392.511975	
diagenode spike set	5703.626851	to	10726.59165	

Table S3 Primer sequence and coordinates for primers used in Table S2.

sox2 FP	tttgcacctgtacctccgaa
sox2 RP	gaaatccacagccactcttg
sox2 coordinates (Zv9)	chr22:40,332,024-40,332,146
tert FP	agacggctacagcaggacag
tert RP	agcgtttagccatgaactcc
tert coordinates (Zv9)	chr19:605,507-605,752
