# Supplementary material for: Genomics of CpG Methylation in Developing and Developed Zebrafish
Source: G3 (Bethesda). 2014 Mar 21;4(5):861–9. doi: 10.1534/g3.113.009514 (PMC4025485; doi:10.1534/g3.113.009514)
Supplement: Supporting Information [file supp_g3.113.009514_FigureS1.pdf]

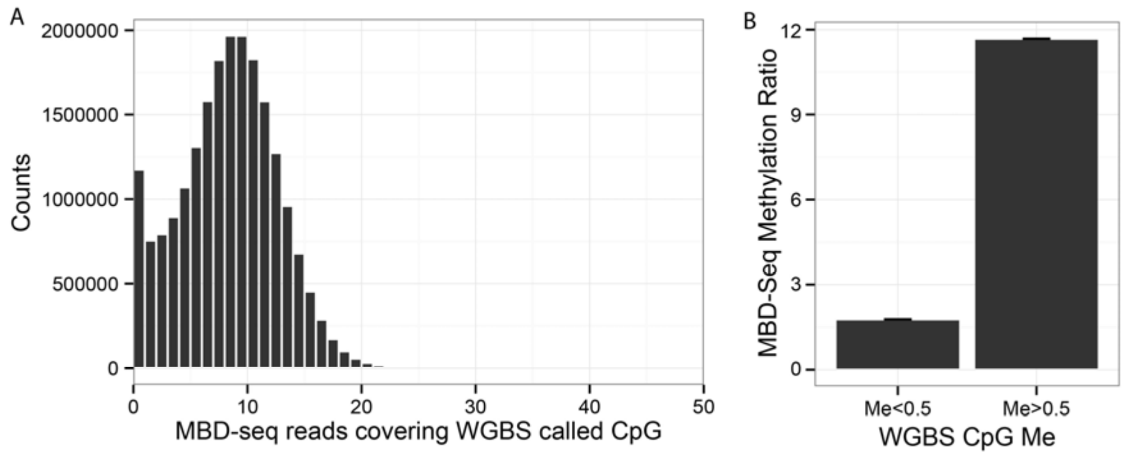

Figure S1 Concordance of MBD-seq and Jiang *et al.* whole genome bisulfite-seq (WGBS) at the MBT time point in zebrafish. (A) Histogram of methylation enriched reads overlapping Jiang et al. identified methylated CpGs. Over 70% of the $19.4x10^6$ CpGs with a methylation of greater than 0.5 (1 being fully methylated, 0 being unmethylated) have at least one overlapping MBD-seq read. (B) Average MBD-seq read ratio scores at Jiang et al. CpGs with methylation less than 0.5 and greater than 0.5. Standard error shown.
