# Supplementary material for: Genomics of CpG Methylation in Developing and Developed Zebrafish
Source: G3 (Bethesda). 2014 Mar 21;4(5):861–9. doi: 10.1534/g3.113.009514 (PMC4025485; doi:10.1534/g3.113.009514)
Supplement: Supporting Information [file supp_g3.113.009514_FigureS2.pdf]

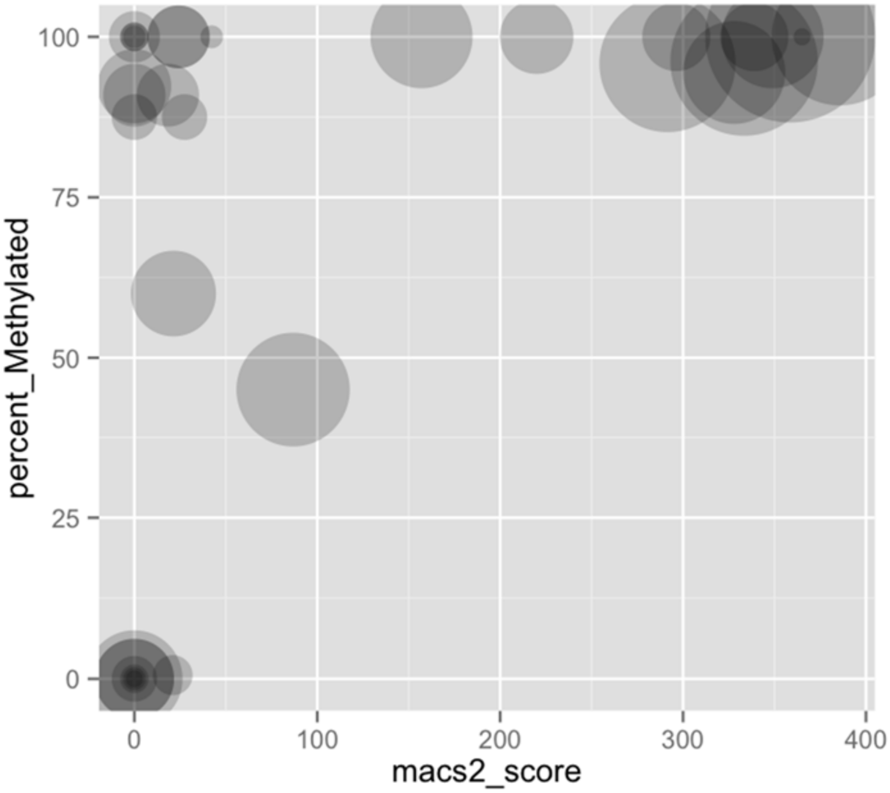

Figure S2 MACS2_score versus bisulfite-converted CpG methylation (sanger sequencing). MACS2 score is on the x-axis with percent CpG methylation on the y-axis, as determined by bisulfite conversion and sanger sequencing. The size of the dot is proportional to the number of CpGs assayed.
