# Supplementary material for: Genomics of CpG Methylation in Developing and Developed Zebrafish
Source: G3 (Bethesda). 2014 Mar 21;4(5):861–9. doi: 10.1534/g3.113.009514 (PMC4025485; doi:10.1534/g3.113.009514)
Supplement: Supporting Information [file supp_g3.113.009514_FigureS3.pdf]

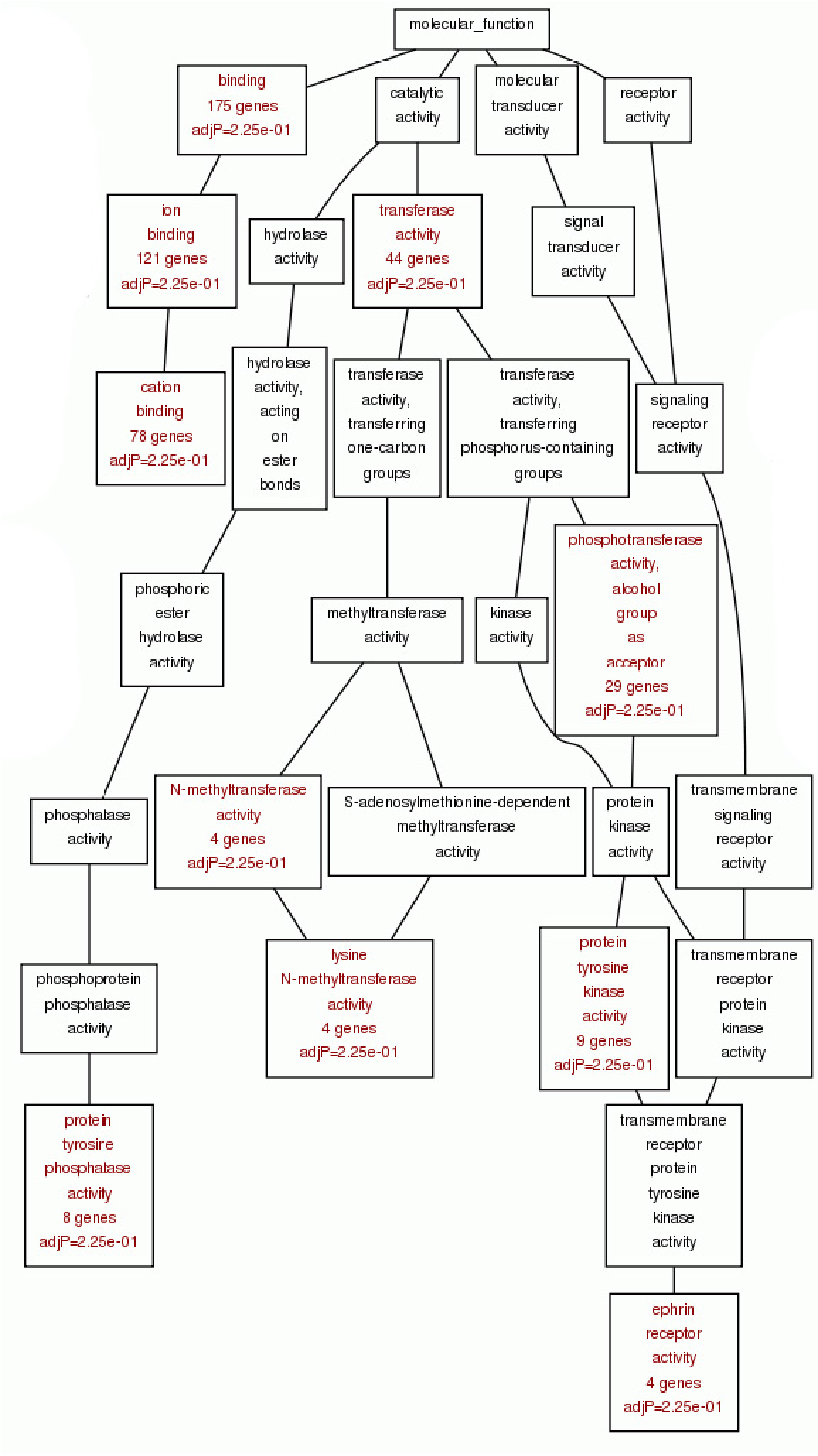

Figure S3 WebGestalt molecular function GO term analysis genes with unique exon peaks in 3dpf methotrexate treated zebrafish relative to control 3dpf zebrafish.
