# Supplementary material for: Genomics of CpG Methylation in Developing and Developed Zebrafish
Source: G3 (Bethesda). 2014 Mar 21;4(5):861–9. doi: 10.1534/g3.113.009514 (PMC4025485; doi:10.1534/g3.113.009514)
Supplement: Supporting Information [file supp_g3.113.009514_TableS1.pdf]

Table S1 Enrichment performance of the NEB EpiMark 5-mC system. Ct scores from qPCR are shown along with the standard deviation. Enrichment is calculated by comparing Ct values from regions known to be methylation positive (me+) to regions known to be methylation poor (me-).

Lane	Tissue	Number of Total Reads	Number of Reads mapQ > 5	Number of Properly Paired Unique Reads, mapQ > 5
4	Brain	87,411,962	40,717,029	17,146,152
4	UnMe Brain (Control)	93,316,244	74,579,958	37,140,721
4	Liver	89,493,944	43,088,933	18,221,225
4	Heart	88,729,694	42,024,288	18,593,167
4	Eye	86,522,756	39,303,576	14,639,460
5	Brain	89,582,450	41,089,919	17,344,084
5	UnMe Brain (Control)	89,959,638	71,824,799	35,933,160
5	1-cell	69,856,440	45,189,947	15,677,629
5	Sperm	75,491,368	42,197,998	19,429,347
5	MBT	63,402,436	36,063,223	17,375,340
3	3dpf.mtx	86,531,596	49,655,034	16,627,123
3	3dpf	85,494,066	45,460,989	19,458,515
3	3dpf.mtx UnMe (Control)	95,881,946	76,243,106	38,340,957
3	3dpf UnMe (Control)	95,881,946	89,661,095	45,038,786
