# Supplementary material for: Genomics of CpG Methylation in Developing and Developed Zebrafish
Source: G3 (Bethesda). 2014 Mar 21;4(5):861–9. doi: 10.1534/g3.113.009514 (PMC4025485; doi:10.1534/g3.113.009514)
Supplement: Supporting Information [file supp_g3.113.009514_TableS2.pdf]

Table S2 Enrichment performance of the NEB EpiMark 5-mC system. Ct scores from qPCR are shown along with the standard deviation. Enrichment is calculated by comparing Ct values from regions known to be methylation positive (me+) to regions known to be methylation poor (me-).

	Me_Enriched		Flow_through (me_poor)	
primers	Ct score	stdev	Ct score	stdev
tert (me+)	21.1439275	0.171582996	22.16167	0.003125412
sox2 (me-)	32.9350965	0.115988847	21.146596	0.331689649
diagenode spike me+	21.6001225	0.356402324	21.750327	0.09125213
diagenode spike me-	32.073724	0.252106195	19.2906445	0.09318041
Enrichment (in Ct)				
tert/sox2	12.806243	0.391052335		
diagenode spike set	12.933284	0.455620116		
Fold Enrichment:				
tert/sox2	5461.913175	to	9392.511975	
diagenode spike set	5703.626851	to	10726.59165	
