# Supplementary material for: Genomics of CpG Methylation in Developing and Developed Zebrafish
Source: G3 (Bethesda). 2014 Mar 21;4(5):861–9. doi: 10.1534/g3.113.009514 (PMC4025485; doi:10.1534/g3.113.009514)
Supplement: Supporting Information [file supp_g3.113.009514_TableS3.pdf]

Table S3 Primer sequence and coordinates for primers used in Table S2.

sox2 FP	tttgcacctgtacctccgaa
sox2 RP	gaaatccacagccactcttg
sox2 coordinates (Zv9)	chr22:40,332,024-40,332,146
tert FP	agacggctacagcaggacag
tert RP	agcgtttagccatgaactcc
tert coordinates (Zv9)	chr19:605,507-605,752
